# Optimal Linear Error Correcting Delivery Schemes for Two Optimal Coded Caching Schemes †

**DOI:** 10.3390/e22070766

**Published:** 2020-07-13

**Authors:** Nujoom Sageer Karat, Anoop Thomas, Balaji Sundar Rajan

**Affiliations:** 1 Department of Electrical Communication Engineering, Indian Institute of Science, Bangalore 560012, India; nujoom@iisc.ac.in; 2 School of Electrical Sciences, Indian Institute of Technology Bhubaneswar, Odisha 752050, India; anoopthomas@iitbbs.ac.in

**Keywords:** coded caching, index coding, error correcting delivery schemes

## Abstract

For coded caching problems with small buffer sizes and the number of users no less than the amount of files in the server, an optimal delivery scheme was proposed by Chen, Fan, and Letaief in 2016. This scheme is referred to as the CFL scheme. In this paper, an extension to the coded caching problem where the link between the server and the users is error prone, is considered. The closed form expressions for average rate and peak rate of error correcting delivery scheme are found for the CFL prefetching scheme using techniques from index coding. Using results from error correcting index coding, an optimal linear error correcting delivery scheme for caching problems employing the CFL prefetching is proposed. Another scheme that has lower sub-packetization requirement as compared to CFL scheme for the same cache memory size was considered by J. Gomez-Vilardebo in 2018. An optimal linear error correcting delivery scheme is also proposed for this scheme.

## 1. Introduction

The problem of coded caching introduced in [1] plays a crucial role in reducing peak hour traffic in networks. In a coded caching scheme, a part of the content is made available in local cache of users, so that traffic can be reduced at peak hours. Coded caching scheme involves two phases: a placement phase and a delivery phase. In the placement phase or the prefetching phase, which is performed during off-peak times, the entire database is made available to each user. Users fill their cache with the available data. Delivery phase is carried out once the demands are revealed by the users. During prefetching, some parts of files have to be judiciously cached at each user in such a way that the rate of transmission is reduced during the delivery phase. The prefetching can be done with or without coding. If during prefetching, no coding of parts of files is done, the prefetching scheme is referred to as uncoded prefetching [1,2]. If coding is done during prefetching stage, then the prefetching scheme is referred to as coded prefetching [3,4,5,6,7].

The seminal work in [1] shows that apart from the local caching gains obtained by placing contents at user caches before the demands are revealed, a global caching gain can be obtained by coded transmissions. This scheme is extended to decentralized scheme in [8]. The caching problem is extended to the case of non-uniform demands [9], online coded caching [10], hierarchical caching [11], and device-to-device caching [12]. Cache management scheme which incorporates decoding information in making cache replacement decisions was studied in [13]. A decentralized secure coded caching approach was proposed in [14], in which nodes only transmit coded files to avoid eavesdropper wiretapping and protect the user contents.

An error correcting delivery scheme is required if the shared bottleneck link between the server and the users is error-prone. The minimum average rate and minimum peak rate of error correcting delivery schemes is characterized in [15]. The placement phase is assumed to be error-free. This assumption can be justified as during the placement phase there is no bandwidth constraint and any number of re-transmissions can be done to make the placement error-free. A similar model in which the delivery phase takes place over a packet erasure broadcast channel was considered in [16,17,18,19].

In this paper, we consider the caching schemes considered in [3,4]. The prefetching scheme employed in these papers is coded, i.e., parts of files are coded and placed in the user caches. Optimal linear error correcting delivery schemes are proposed for these prefetching schemes. The optimal error correcting delivery scheme refers to an error correcting delivery scheme, which uses the minimum number of transmissions for a given placement. The main contributions of this paper are as follows.

- The prefetching scheme proposed in [3] is considered for the case where the number of users is the same as the number of files. The minimum number of transmissions required for correcting finite number of transmission errors is obtained for this case (Section 3.1).
- When the number of users is greater than the number of files, a different prefetching scheme is employed in [3]. For this prefetching strategy, the minimum number of transmissions required for correcting finite number of transmission errors is obtained (Section 3.2).
- A linear error correcting delivery scheme for coded caching problem with coded prefetching for small buffer sizes is proposed. The expressions for average rate and peak rate of this error correcting delivery scheme are found (Section 4).
- The caching scheme in [4] is considered for the same cache memory region. The advantage here is that the sub-packetization requirement is low. Sub-packetization level refers to number of subfiles that each file is split into. An optimal linear error correcting delivery scheme is found for the prefetching scheme employed in [4] (Section 5).

Parts of the content of this manuscript are present in [20]. The additional content in this manuscript is the construction of optimal linear error correcting delivery scheme for the prefetching scheme employed in [4] with reduced sub-packetization (Section 5). The proofs of Theorem 1 and Theorem 2 are also not present in [20].

A work that is closely related to this work is present in [15] and its extended version [21]. In [21], error correction is considered for a particular class of uncoded prefetching, namely symmetric batch prefetching. The major distinctions of this paper with the work in [21] are as follows. This is the first paper in the literature that considers error correction for coded caching schemes with coded prefetching. On the other hand, the work in [21] involves uncoded prefetching. For a fixed demand, the delivery scheme of a coded caching problem with uncoded prefetching is an index coding problem [22]. When the prefetching is coded, it corresponds to a generalized index coding problem [23]. To derive bounds in [21], the concepts of index coding are used and, to derive the bounds in this paper, we use techniques from generalized index coding. This paper is the first to use the concepts of generalized index coding to derive bounds for coded caching problems.

In this paper, $F_{q}$ denotes the finite field with *q* elements, where *q* is prime power, and $F_{q}^{*}$ denotes the set of all non-zero elements of $F_{q}$. For any positive integer *K*, [K] denotes the set {1,2,…,K}. For a K×N matrix L, $l_{i}$ denotes its *i*th row. For vector spaces U,V, U<V denotes that U is a subspace of V. A linear ${\left[n,k,d\right]}_{q}$ code C over $F_{q}$ is a *k*-dimensional subspace of $F_{q}^{n}$ with minimum Hamming distance *d*. The vectors that belong to the subspace C are called codewords. A matrix G of size k×n whose rows are linearly independent codewords of C is called a generator matrix of C. Thus, a linear ${\left[n,k,d\right]}_{q}$ code C can be represented using its generator matrix G as, $C=\{yG:y\in F_{q}^{k}\}.$ $N_{q}\left[k,d\right]$ denotes the length of the shortest linear code over $F_{q}$, which has dimension *k* and minimum distance *d*.

## 2. Preliminaries and Background

Results from error correction for index coding with coded side information are used to obtain error correcting delivery schemes for the caching problem. In this section we recall results from error correction for index coding with coded side information introduced in [24]. We also review the coded caching scheme with coded prefetching proposed in [3,4], for which optimal error correcting delivery schemes are presented in the following sections (Section 3, Section 4, and Section 5).

### 2.1. Generalized Index Coding Problem and Error Correction

The index coding (IC) problem with side-information was introduced by Birk and Kol [22]. A source broadcasts messages through a noiseless shared channel to multiple receivers, each demanding certain messages and knowing some other messages a priori as side-information. The source needs to meet the demands of each receiver in a minimum number of transmissions. The minimum number of transmissions required to meet the demands of all the receivers is termed as the optimal length of the index coding problem. In [23] and [25], a generalization of the index coding problem was discussed, where the demands of the receivers and the side-information are linear combinations of the messages. In [25], the authors refer to this class of problems as Generalized Index Coding (GIC) problems.

An instance, I of a GIC problem is described formally, as follows. There is a message vector $x^{T}={\left(x_{1},x_{2},\ldots ,x_{n}\right)}^{T}\in F_{q}^{n\times 1}$ and there are *m* receivers. The *i*th receiver demands a linear combination of the messages $r_{i}x^{T}$, for some $r_{i}\in F_{q}^{1\times n}$, where $r_{i}$ is the request vector and $r_{i}x^{T}$ is the request packet of the *i*th receiver. The side-information is represented by a matrix $V^{\left(i\right)}\in F_{q}^{s_{i}\times n}$, where $s_{i}$ is the number of packets possessed as side-information by the *i*th receiver. The packets that are known as side-information possessed by the *i*th receiver are $V^{\left(i\right)}x^{T}$. Let $X^{\left(i\right)}$ denote the row space of $V^{\left(i\right)}$. Receiver *i* can also compute the linear combination of messages $vx^{T}$$\forall v\in X^{\left(i\right)}$. Let R be an m×n matrix over $F_{q}$ having $r_{i}$ as its *i*th row. The matrix R represents the demands of all the *m* receivers. In the definition of GIC problem in [25] the source is assumed to possess only certain linear combinations of messages. In our work, it is assumed that all of the messages are independent and the source possesses all of them.

The min-rank of an instance I of the GIC problem over $F_{q}$ is defined as

$$\kappa \left(I\right)=\min\{\mathrm{rank}\left(A+R):A\in F_{q}^{m\times n},a_{i}\in X^{\left(i\right)},i\in \left[m\right]\right\}.$$

This is motivated by the fact that any encoding matrix for a GIC problem is of the form A+R. It is shown in [24] that the min-rank is the optimal length of linear generalized index code. Intuitively, min-rank can be viewed as the minimum communication load among all of the delivery schemes with linear decoding functions, where each $a_{i}$ corresponds to the coefficients for the locally cached contents.

For each i∈[m], the set $Z^{\left(i\right)}$ is defined as

(1) $$Z^{\left(i\right)}≜\left\{z\in F_{q}^{n\times 1}:V^{\left(i\right)}z=0,r_{i}z\neq 0\right\}.$$

Thus, $Z^{\left(i\right)}$ is a collection of vectors that belong to the null space of $V^{\left(i\right)}$ and satisfy $r_{i}z\neq 0$. The set of vector spaces J(I) is defined as $J\left(I\right)≜\{U<F_{q}^{n}:U\setminus \left\{0\right\}\subset \cup_{i\in [m]}Z^{\left(i\right)}\}$. The set J(I) is the collection of subspaces such that the set of non-zero vectors of any subspace in the collection is a subset of $\cup_{i\in [m]}Z^{\left(i\right)}$. The maximum value of dimension among the dimensions of all the elements of J(I) is called the generalized independence number, denoted by α(I). Thus, the dimension of any subspace of $F_{q}^{n}$ in $\cup_{i\in [m]}Z^{\left(i\right)}\cup \left\{0\right\}$ serves as a lower bound for α(I). The generalized independence number can be viewed as a cut set type of argument when the message lives in a subspace where all of the cached contents are zero. It was shown in [24] that the min-rank serves as an upper bound for the generalized independence number, i.e.,

(2) α(I)≤κ(I).

Generalized index coding problems were classified in [26]. In generalized index coding with coded side-information problems, the demand of every receiver is uncoded but the side-information is coded. In generalized index coding problems with coded demands, the side-information of every receiver is uncoded but the demand is coded. In our work the focus is on generalized index coding with coded side-information problems.

Error correcting index codes were introduced in [27] and later extended for generalized index coding problems in [24]. An Error Correcting Generalized Index Code (ECGIC) is a map that encodes the message vector $x^{T}$, such that each user, given its side-information and received transmissions, can decode its requested packet $r_{i}x^{T}\in F_{q}$, even in the presence of at most δ transmission errors. The smallest possible length of such a δ-error correcting index code is denoted by $N_{q}\left[I,\delta \right]$. An optimal linear (I,δ)-ECGIC over $F_{q}$ is a linear (I,δ)-ECGIC over $F_{q}$ of the smallest possible length. The optimal length of a linear error correcting index code is lower bounded by α-bound and upper bounded by κ-bound as

(3) $$\underset{\alpha -\mathrm{bound}}{\underset{︸}{N_{q}\left[\alpha \left(I\right),2\delta +1\right]}}\;\leq \;N_{q}\left[I,\delta \right]\;\leq \;\underset{\kappa -\mathrm{bound}}{\underset{︸}{N_{q}\left[\kappa \left(I\right),2\delta +1\right]}}.$$

where $N_{q}\left[k,d\right]$ is the length of an optimal linear classical error-correcting code of dimension *k* and minimum distance *d* over $F_{q}$ [24,27].

If for some index coding problem, α(I)=κ(I), then bounds in (3) meet with equality. Thus, for such problems,

$$N_{q}\left[\alpha \left(I\right),2\delta +1\right]=N_{q}\left[I,\delta \right]=N_{q}\left[\kappa \left(I\right),2\delta +1\right].$$

In general, the κ-bound is obtained by concatenating an optimal linear classical error correcting code and an optimal linear index code. When α(I)=κ(I), as the optimal length of the ECGIC is same as κ-bound, for such problems, concatenation scheme would give optimal linear error correcting index codes [26,28,29,30].

### 2.2. Error Correcting Coded Caching Scheme

The problem of error correcting coded caching scheme was proposed in [15]. The server is connected to *K* users through a shared link, which is error prone. The server has access to *N* files $X_{1},X_{2},\ldots ,X_{N}$ each of size *F* bits. Every user has an isolated cache with memory MF bits, where M∈[0,N]. There are many ways in which users can fill their cache contents. A prefetching scheme is denoted by M and it specifies the way in which user caches are filled. Each user demands one of the *N* files. Let the demand vector be $d=(d_{1},\ldots ,d_{K})$, where $d_{i}$ is the index of the file demanded by user *i*. The number of distinct files requested in d is denoted by $N_{e}\left(d\right)$. The set of all possible demands is denoted by $D={\left\{1,2,\ldots ,N\right\}}^{K}.$ During the delivery phase, the server informed of the demand d, transmits a function of $X_{1},X_{2},\ldots ,X_{N}$ over a shared link. A δ-error correcting coded caching scheme should be in such a way that using the cache contents and the transmitted data, each user *i* needs to decode the requested file $X_{d_{i}}$, even if δ transmissions are in error.

For a δ-error correcting coded caching problem, a communication rate R(δ) is achievable for demand d if and only if there exists a transmission of R(δ)F bits such that every user *i* is able to decode its desired file $X_{d_{i}}$ even after at most δ transmissions are in error. Rate $R^{*}\left(d,M,\delta \right)$ is the minimum achievable rate for a given d, M and δ. The average rate $R^{*}\left(M,\delta \right)$ is defined as the expected minimum average rate for a given M and δ under uniformly random demand. Thus, $R^{*}\left(M,\delta \right)=E_{d}\left[R^{*}\left(d,M,\delta \right)\right].$ Another quantity of interest is the peak rate, denoted by $R_{\mathrm{worst}}^{*}\left(M,\delta \right)$, which is defined as $R_{\mathrm{worst}}^{*}\left(M,\delta \right)=\max_{d}R^{*}\left(d,M,\delta \right).$

### 2.3. Coded Caching Scheme with Coded Prefetching

An optimal coded caching scheme for small cache sizes involving coded prefetching was given in [3] by Chen, Fan, and Letaief. We refer this scheme as the Chen Fan Letaief (CFL) scheme. The prefetching scheme here is denoted by $M_{\mathrm{CFL}}$. For this scheme, the cache memory size of each user is M=1/K. The prefetching strategy differs depending on the values of *N* and *K*. Thus, $M_{\mathrm{CFL}}$ involves two types of prefetching strategies, which are as given below:

- Consider the case when N=K and M=1/K. Each file is split into *N* subfiles, i.e., $X_{i}=\left(X_{i,1},X_{i,2},\ldots ,X_{i,N}\right).$ During prefetching, the cache of user *j* is filled as $Y_{j}=X_{1,j}⊕X_{2,j}⊕\ldots ⊕X_{N,j}$, an XORed version of subfiles. It is shown in [3] that $R\left(\delta =0\right)=N_{e}\left(d\right)$ for $N_{e}\left(d\right)\leq N-1$ and R(δ=0)=N−1 for $N_{e}\left(d\right)=N$ are achievable. Furthermore, if M∈[0,1/N],$R^{*}\left(M,\delta =0\right)=N\left(1-M\right)$ is achievable by memory sharing.
- Consider K>N and M=1/K. Each file is split into NK subfiles, i.e., $X_{i}=(X_{i,1},X_{i,2},$$\ldots ,X_{i,NK}).$ The cache of user *i* is given by $Y_{i}=X_{1,N(i-1)+j}⊕\ldots ⊕X_{N,N(i-1)+j},$ for j=1,2,…,N. For the number of distinct demands $N_{e}\left(d\right)\leq N-1$ files, it is shown in [3] that $R\left(\delta =0\right)=N_{e}\left(d\right)$ is achievable. For $N_{e}\left(d\right)=N$, the rate R(δ=0)=N−N/K is achievable. Furthermore, if M∈[0,1/K],$R^{*}\left(M,\delta =0\right)=N\left(1-M\right)$ is achievable by memory sharing.

### 2.4. Coded Caching Scheme with Coded Prefetching with Low Sub-Packetization

A scheme for N≤K and cache capacities 1/K≤M≤N/K was proposed in [4] by Jesus Vilardebo. We refer to this scheme as the Jesus Vilardebo (JV) scheme. The prefetching scheme of the JV scheme is denoted by $M_{\mathrm{JV}}$. We consider this scheme only for M=1/K. The advantage of JV scheme over CFL scheme is that the sub-packetization requirement is low. The scheme in [4] uses a low sub-packetization level as compared to the scheme in [3]. A low sub-packetization level is always preferred, because any practical scheme will require each of the subfiles to have some header information that allows for decoding at the end users. When there are a large number of subfiles, the header overhead may be non-negligible. The prefetching scheme, $M_{\mathrm{JV}}$, is described, as follows: each file $X_{i}$ is partitioned into *K* parts $X_{i,1},X_{i,2},\ldots ,X_{i,K}$ for i∈[N]. Afterwards, the cache content for user *i* is populated as $Y_{i}:X_{1,i}⊕X_{2,i}⊕\ldots ⊕X_{N,i}$ for i∈[K]. The rate achieved is same as the CFL scheme.

### 2.5. Index Coding and Coded Caching

For a fixed prefetching M and for a fixed demand d, the delivery phase of a coded caching problem is an index coding problem [1]. We denote such an index coding problem as I(M,d). In fact, for fixed prefetching, a coded caching scheme consists of $N^{K}$ parallel index coding problems one for each of the $N^{K}$ possible user demands. Thus, finding the minimum achievable rate for a given demand d is equivalent to finding the min-rank of the equivalent index coding problem induced by the demand d. Because the generalized independence number and min-rank of I(M,d) depend on the caching scheme M and demand d, we denote them as α(M,d) and κ(M,d), respectively.

Consider the CFL prefetching scheme $M_{\mathrm{CFL}}$. The index coding problem that is induced by the demand d for the CFL prefetching is $I(M_{\mathrm{CFL}},d).$ Each subfile $X_{i,j}$ corresponds to a message in the index coding problem. Because prefetching is coded, $I(M_{\mathrm{CFL}},d)$ represents a generalized index coding with coded side-information problem. Similarly, $I(M_{\mathrm{JV}},d)$ represents a generalized index coding problem that corresponds to the JV prefetching scheme.

There have been many works in the literature that make use of the link between index coding and coded caching. Some of them use index coding concepts to derive lower bounds on the rate [31,32]. In [33], multiple groupcast index coding is used to design coded caching delivery for multiple requests. A novel index coding scheme was introduced in [34], which, when applied to caching problem, is then shown to match an outer bound under the assumption of uncoded cache prefetching. A multihop index coding technique is proposed in [35] to code the cached contents in helpers to achieve order-optimal capacity gains. Decentralized caching schemes were proposed for two-layer networks in [36] which exploit index coding in the delivery phase and leverages multicast opportunities. None of these assume the channel to be error prone during the delivery phase. In our work, we use the error correction aspects of index coding to design optimal error correcting coded caching schemes.

We call a caching scheme optimal if both the placement and delivery schemes are designed in such a way to achieve an optimal rate memory pair. An optimal error correcting delivery scheme refers to an error correcting delivery scheme, which uses the minimum number of transmissions for a given placement. Unless the delivery scheme is carefully designed, one may end up with non-optimal error correcting delivery scheme for a given placement. This is clear from Example 4.8 of [27]. In that example, an index coding problem with five messages and five receivers are considered. The side information sets are given as $X_{1}=\left\{2,5\right\}$, $X_{2}=\left\{1,3\right\}$, $X_{3}=\left\{2,4\right\}$, $X_{4}=\left\{3,5\right\}$, and $X_{5}=\left\{1,4\right\}$. For this problem, it can be calculated that α(I)=2. Additionally, for this problem, min-rank, κ(I)=3 over binary field. From code tables in [37], we have $N_{2}\left[2,5\right]=8$ and $N_{2}\left[3,5\right]=10.$ Hence, $8\leq N_{2}\left[I,\delta \right]\leq 10$. Using a computer search, the authors of [27] have found that the optimal length $N_{2}\left[I,2\right]=9$. Here the optimal length of the error correcting index code lies strictly between the α-bound and the κ-bound. Thus, for this problem, the construction of optimal linear error correcting index code by concatenation is not optimal. Similarly, in general for coded caching problems with an arbitrary placement and demand, the optimal error correcting delivery scheme cannot be constructed by concatenation unless we prove that the α and κ bounds meet for the corresponding index coding problem. In the schemes which we consider in this paper, we explicitly prove that the α and κ bounds meet for all of the demand cases for the given placement. Hence, the optimality of concatenation is guaranteed.

## 3. Generalized Independence Number for $I(M_{\mathrm{CFL}},d)$

In this section we find a closed form expression for the generalized independence number $\alpha (M_{\mathrm{CFL}},$ d) of the index coding problem $I(M_{\mathrm{CFL}},d)$. There are two different prefetching schemes employed in [3], depending on the relationship between the number of messages and the number of receivers. For all the index coding problems corresponding to both these prefetching schemes, the generalized independence number is shown to be equal to the min-rank.

### 3.1. Number of Files Equal to the Number of Users (N=K)

In the CFL prefetching scheme, each file is split into *N* subfiles. Hence, the number of messages in $I(M_{\mathrm{CFL}},d)$ is $N^{2}$. Each user is split into *N* receivers each demanding one message. Hence, there are a total of $N^{2}$ receivers. From the expressions of the achievable rates in [3], we get the min-rank $\kappa (M_{\mathrm{CFL}},d)$ as

(4) $$\kappa \left(M_{\mathrm{CFL}},d\right)\leq \left\{\begin{array}{cc} N_{e}\left(d\right)N & \mathrm{if}\;N_{e}\left(d\right)\leq N-1\\ N(N-1) & \mathrm{if}\;N_{e}\left(d\right)=N \end{array}\right..$$

We find the generalized independence number $\alpha (M_{\mathrm{CFL}},d)$ for $I(M_{\mathrm{CFL}},d)$. For different demands, a generalized independence number is calculated and it is shown to be equal to the min-rank of the corresponding generalized index coding problem. The technique of obtaining $\alpha (M_{\mathrm{CFL}},d)$ is illustrated in the following example.

**Figure 1:**
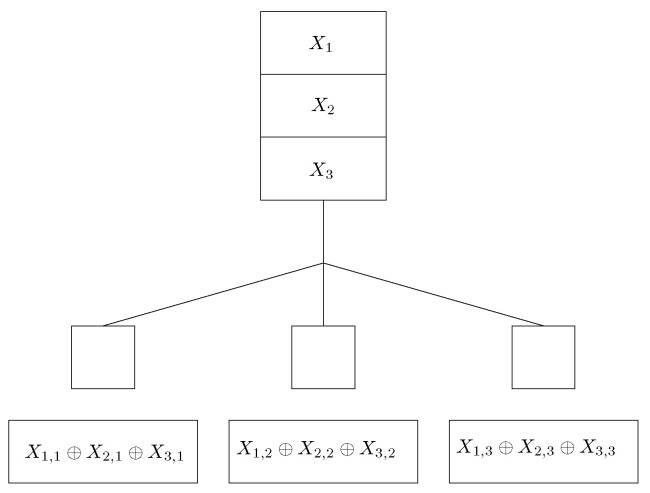
Coded Caching problem with N=K=3, M=1/3 and the hen Fan Letaief (CFL) placement.

**Example** **1.** *Consider a coded caching problem with N=K=3, M=1/3 (see Figure 1). Because M=1/K, the CFL scheme is used for solving the coded caching problem. Each file is split into N=3 subfiles as $X_{1}=\left(X_{1,1},X_{1,2},X_{1,3}\right)$, $X_{2}=\left(X_{2,1},X_{2,2},X_{2,3}\right)$ and $X_{3}=\left(X_{3,1},X_{3,2},X_{3,3}\right)$. Let $X=(X_{1,1},X_{1,2},X_{1,3},X_{2,1},X_{2,2},X_{2,3},X_{3,1},X_{3,2},X_{3,3})$ denote the vector obtained by concatenating $X_{1},X_{2}$ and $X_{3}$. The cache contents of user i is $Y_{i}=\left(X_{1,i}⊕X_{2,i}⊕X_{3,i}\right)$ for i=1,2,3. Cache contents are depicted in Figure 1.*

*First consider that all of the demands are distinct, i.e., $N_{e}\left(d\right)=3$. Without loss of generality, we can assume that the demand is d=(1,2,3). Consider the equations*

$$\begin{array}{cc} e_{1} & :X_{1,1}⊕X_{2,1}⊕X_{3,1}=0,\\ e_{2} & :X_{1,2}⊕X_{2,2}⊕X_{3,2}=0,\;and\;\\ e_{3} & :X_{1,3}⊕X_{2,3}⊕X_{3,3}=0. \end{array}$$

*Let S be the subspace of $F_{q}^{9}$, which consists of the vectors satisfying the equations $e_{1},e_{2}$ and $e_{3}$. From the rank-nullity theorem, dim(S)≥6. The induced generalized index coding problem $I(M_{\mathrm{CFL}},d)$ has 9 messages and 9 receivers. For this case, (1) can be rewritten as $Z^{\left(i,j\right)}≜\left\{z\in F_{q}^{9}:e_{i},X_{d_{i},j}\neq 0\right\}.$ Let $A\left(d\right)=\cup_{i,j\in [3]}Z^{\left(i,j\right)}\cup \left\{0\right\}$. The generalized independence number is the maximum among the dimensions of all the subspaces of $F_{q}^{9}$ in A(d). We claim that all the vectors of S belong to the set A(d). Thus, $\alpha \left(M_{\mathrm{CFL}},d\right)\geq \dim\left(S\right)\geq 6$. From the definition of A(d), it is clear that the all zero vectors 0 belonging to S also belong to A(d). Any other vector in S will have at least one non-zero coordinate $X_{i,j}$. The vector belonging to S, having $X_{i,j}\neq 0$ belongs to the set $Z^{\left(i,j\right)}$. Thus, all of the vectors in S lie in A(d) and $\alpha (M_{\mathrm{CFL}},d)\geq 6.$ From (4), we get $\kappa (M_{\mathrm{CFL}},d)\leq 6$. Hence, by (2), we have $\alpha \left(M_{\mathrm{CFL}},d\right)=\kappa \left(M_{\mathrm{CFL}},d\right)=6.$*

*Finally, assume $N_{e}\left(d\right)=1$ and let d=(1,1,1). In addition to $e_{1},e_{2}$ and $e_{3}$, consider the following set of equations $e_{4}:X_{2,1}=0,$$e_{5}:X_{2,2}=0,\;\mathrm{and}\;$$e_{6}:X_{2,3}=0.$ Let S be the subspace of $F_{q}^{9}$, which consists of the vectors that satisfy the set of equations $e_{1},e_{2},\ldots ,e_{6}.$ We follow the similar argument as above to show that all the vectors in S lie in A(d). By definition, 0 lies in A(d). All of the vectors in S with $X_{1,j}\neq 0$ for j=1,2,3 are present in A(d). By $e_{4},e_{5}$ and $e_{6}$, all the vectors in S have $X_{2,j}=0$. The condition $X_{3,j}\neq 0$ and the set of equations $e_{1},e_{2},\ldots ,e_{6}$ force $X_{1,j}\neq 0$. Hence, all of the vectors in S with $X_{3,j}\neq 0$ are present in $Z^{\left(1,j\right)}$. Thus, all of the vectors in S are present in A(d). Moreover, dim(S)≥9−6=3. Therefore $\alpha (M_{\mathrm{CFL}},d)\geq 3.$ From (4), $\kappa (M_{\mathrm{CFL}},d)\leq 3$. Thus, by (2), $\alpha \left(M_{\mathrm{CFL}},d\right)=\kappa \left(M_{\mathrm{CFL}},d\right)=3.$ For other possible demands also, it can be verified from Table 1 that $\alpha \left(M_{\mathrm{CFL}},d\right)=\kappa \left(M_{\mathrm{CFL}},d\right)$.*

In Example 1, the generalized independence number of the index coding problem $I(M_{\mathrm{CFL}},d)$ is equal to its min-rank. For different demands, the generalized index coding problem changes and, for all those problems, min-rank and generalized independence number are shown to be equal. This can be shown for all values of *N*, as given in the theorem below.

**Theorem** **1.** *For N=K and M=1/K,*

$$\begin{array}{cc} \alpha (M_{\mathrm{CFL}},d) & =\kappa (M_{\mathrm{CFL}},d)\\ & =\left\{\begin{array}{cc} N_{e}\left(d\right)N & if\;N_{e}\left(d\right)\leq N-1\\ N(N-1) & if\;N_{e}\left(d\right)=N \end{array}\right., \end{array}$$

*∀d∈D, where $N_{e}\left(d\right)$ is the number of distinct demands.*

**Proof.**  In the CFL prefetching scheme $M_{\mathrm{CFL}}$, each file $X_{i},i\in \left[N\right]$ is split into *N* subfiles $X_{i,1},X_{i,2},\ldots ,X_{i,N}$. User i,i∈[N] caches $Y_{i}=\left(X_{1,i}⊕X_{2,i}⊕\ldots ⊕X_{N,i}\right)$. Let $X=(X_{1,1},\ldots ,X_{1,N},X_{2,1},\ldots ,X_{2,N},\ldots ,X_{N,1},\ldots ,X_{N,N})$ be the vector obtained by concatenation of vectors $X_{i},i\in \left[N\right]$.

For a given demand d, the delivery phase of the coded caching problem becomes a generalized index coding problem $I(M_{\mathrm{CFL}},d)$ with $N^{2}$ messages and $N^{2}$ receivers.

First, consider that all of the demands are distinct, i.e., $N_{e}\left(d\right)=N$. Let the demand of the *i*th user be $X_{d_{i}}$. Thus $d=(d_{1},d_{2},\ldots ,d_{N})$. Consider the set of *N* equations denoted by $e_{1},e_{2},\ldots ,e_{N}$, where

$$e_{i}:\left(X_{1,i}⊕X_{2,i}⊕\ldots ⊕X_{N,i}\right)=0.$$

Let S be the subspace of $F_{q}^{N^{2}}$, which consists of the vectors that satisfy the set of equations $e_{1},e_{2},\ldots ,e_{N}$. From the rank-nullity theorem, we have dim$\left(S\right)\geq N^{2}-N$.

For $I(M_{\mathrm{CFL}},d)$, from (1) we have, $Z^{\left(i,j\right)}≜\left\{z\in F_{q}^{N^{2}}:e_{i},X_{d_{i},j}\neq 0\right\}.$ Let $A\left(d\right)=\cup_{i,j\in [N]}Z^{\left(i,j\right)}\cup \left\{0\right\}$. The generalized independence number is the maximum of the dimensions of all subspaces of $F_{q}^{N^{2}}$ in A(d). We show that S is such a subspace. For this, we need to show that all the vectors of S lie in A(d). By the definition of A(d), the all zero vector 0 lies in A(d). Any other vector in S will have at least one non-zero coordinate. The vectors that belong to S having $X_{d_{i},j}\neq 0$ belongs to the set $Z^{\left(i,j\right)}$. Thus, all of the vectors in S lie in A(d). The generalized independence number $\alpha \left(M_{\mathrm{CFL}},d\right)\geq N^{2}-N.$ From (4), we get $\kappa \left(M_{\mathrm{CFL}},d\right)\leq N^{2}-N$. Hence, by (2), we have $\alpha \left(M_{\mathrm{CFL}},d\right)=\kappa \left(M_{\mathrm{CFL}},d\right)=N^{2}-N.$

Consider the case where $N_{e}\left(d\right)\leq N-1$. Without a loss of generality, we can assume that the first $N_{e}\left(d\right)$ users have distinct demands and that the *i*th user demands the file $X_{d_{i}}$ for $i\in [N_{e}\left(d\right)]$. Also, without loss of generality, we can assume that the set of indices of the files that are not demanded are $N_{e}\left(d\right)+1,N_{e}\left(d\right)+2,\ldots ,N$. There are $U=N-N_{e}\left(d\right)$ files that are not demanded. In addition to $e_{1},e_{2},\ldots ,e_{N}$, consider the following set of equations $X_{N_{e}\left(d\right)+i,j}=0$, for i∈[U−1],j∈[N]. The number of these equations is thus $N+N\left(U-1\right)=NU=N(N-N_{e}\left(d\right))$. Let S be the subspace of $F_{q}^{N^{2}}$, which consists of vectors that satisfy these equations. Hence, dim$\left(S\right)\geq N^{2}-N\left(N-N_{e}\left(d\right)\right)=N_{e}\left(d\right)N.$ By definition, 0 lies in A(d). Any vector with the coordinate $X_{d_{i},j}\neq 0$ for $i\in [N_{e}\left(d\right)]$ lies in $Z^{\left(i,j\right)}$. The set of equations force all $X_{i,j}=0$ for $i\in \{N_{e}\left(d\right),\ldots ,N-1\}$. Moreover if $X_{N,j}\neq 0$ the set of equations force some $X_{d_{i},j}\neq 0$ for some $i\in [N_{e}\left(d\right)]$. Hence any vector with $X_{N,j}\neq 0$ lies in some $Z^{\left(i,j\right)}$ for $i\in [N_{e}\left(d\right)]$. Thus, all of the vectors in S lie in A(d). Therefore, $\alpha \left(M_{\mathrm{CFL}},d\right)\geq \dim\left(S\right)\geq N_{e}\left(d\right)N.$ Applying (4) and (2), $\alpha \left(M_{\mathrm{CFL}},d\right)=\kappa \left(M_{\mathrm{CFL}},d\right)=N_{e}\left(d\right)N.$ □

### 3.2. Number of Users More Than the Number of Files (N<K)

In the CFL prefetching scheme for N<K, each file is split into NK subfiles. Hence, the number of messages in $I(M_{\mathrm{CFL}},d)$ is $N^{2}K$. Each user is split into NK receivers in $I(M_{\mathrm{CFL}},d)$, each demanding a single message. Thus, there are a total of $NK^{2}$ receivers. From the expressions for achievable rates in [3], we obtain the min-rank $\kappa (M_{\mathrm{CFL}},d)$ as

(5) $$\kappa \left(M_{\mathrm{CFL}},d\right)\leq \left\{\begin{array}{cc} NKN_{e}\left(d\right) & \mathrm{if}\;N_{e}\left(d\right)\leq N-1\\ N^{2}\left(K-1\right) & \mathrm{if}\;N_{e}\left(d\right)=N \end{array}\right..$$

We find the generalized independence number $\alpha (M_{\mathrm{CFL}},d)$ for $I(M_{\mathrm{CFL}},d)$. The technique of obtaining $\alpha (M_{\mathrm{CFL}},d)$ is illustrated in the following example.

**Figure 2:**
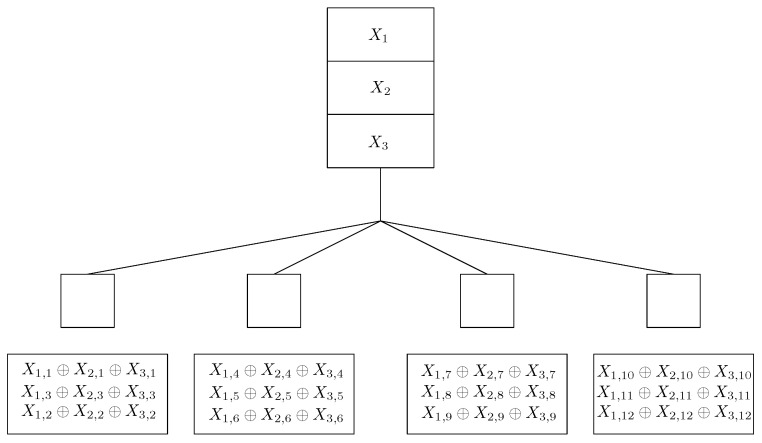
Coded Caching problem with N=3, K=4, M=1/4 and CFL placement.

**Example** **2.** *Consider a coded caching problem with N=3, K=4 and M=1/4 (see Figure 2). According to the CFL scheme each file is split into NK=12 subfiles as $X_{1}=\left(X_{1,1},X_{1,2},\ldots ,X_{1,12}\right)$, $X_{2}=\left(X_{2,1},X_{2,2},\ldots ,X_{2,12}\right)$, and $X_{3}=\left(X_{3,1},X_{3,2},\ldots ,X_{3,12}\right)$. Let $X=(X_{1,1},\ldots ,X_{1,12},\ldots ,X_{3,1},\ldots ,X_{3,12})$ denote the vector obtained by concatenating $X_{1},X_{2}$ and $X_{3}$. The cache of the ith user contains three coded packets $Y_{i}=\left(X_{1,3(i-1)+j}⊕X_{2,3(i-1)+j}⊕X_{3,3(i-1)+j}\right)$ for j=1,2,3. The cache contents are given in Figure 2.*

*For a given demand d, this problem becomes a generalized index coding problem $I(M_{\mathrm{CFL}},d)$,having 36 messages and 48 receivers.*

*First consider that $N_{e}\left(d\right)=N=3$ and d=(1,2,3,1). Consider the nine equations given by $e_{i,j}:\left(X_{1,3(i-1)+j}⊕X_{2,3(i-1+j)}⊕X_{3,3(i-1)+j}\right)=0$ for i=1,2,3 and j=1,2,3. Let S be the subspace of $F_{q}^{36}$ satisfying these nine equations. From the rank-nullity theorem, we get dim(S)≥36−9=27. For this case, (1) can be rewritten as $Z^{\left(i,j\right)}≜\left\{z\in F_{q}^{36}:e_{i,1},e_{i,2},e_{i,3},X_{d_{i},j}\neq 0\right\}$ for i∈[4]. Let $A\left(d\right)=\cup_{i\in [4],j\in [12]}Z^{\left(i,j\right)}\cup \left\{0\right\}$. The generalized independence number is the maximum among the dimensions of all the subspaces of $F_{q}^{36}$ in A(d). We claim that S is such a subspace. This would mean that $\alpha \left(M_{\mathrm{CFL}},d\right)\geq \dim\left(S\right)\geq 27$. For this, we need to show that all of the vectors in S lie in A(d). By the definition of A(d), the all zero vector 0 lies in A(d). Any other vector in S will have at least one non-zero coordinate. All of the vectors in S, having $X_{d_{i},j}\neq 0$ belongs to $Z^{\left(i,j\right)}$. Thus, all of the vectors in S lie in A(d) and $\alpha (M_{\mathrm{CFL}},d)\geq 27.$ From (5), we get $\kappa \left(M_{\mathrm{CFL}},d\right)\leq 3^{2}\left(4-1\right)=27$. Hence, by (2), we have $\alpha \left(M_{\mathrm{CFL}}\right)=\kappa \left(M_{\mathrm{CFL}}\right)=27.$*

*Consider now that $N_{e}\left(d\right)=2$ and d=(1,2,1,2). In addition to the nine equations $e_{i,j}$ for i=1,2,3 and j=1,2,3, consider three more equations $e_{4,j}:\left(X_{1,9+j}⊕X_{2,9+j)}⊕X_{3,9+j}\right)=0$ for j=1,2,3. Thus, we consider a set of twelve equations given by $E=\{e_{i,j}:i\in \left[4\right],j\in \left[3\right]\}$. Let S be the subspace of $F_{q}^{36}$ consisting of vectors that satisfy the equations in E. Hence, from the rank-nullity theorem, we have dim(S)≥36−12=24. By definition, 0 lies in A(d). Any non-zero vector in S with $X_{d_{i},j}\neq 0$ for i=1,2 lies in the corresponding $Z^{\left(i,j\right)}$. By E, any $X_{3,j}\neq 0$ forces some $X_{i,j}\neq 0$ for i=1,2 and, hence, such vectors also lie in A(d). Thus all of the vectors in S lie in A(d). Therefore $\alpha \left(M_{\mathrm{CFL}},d\right)\geq \dim\left(S\right)\geq 24.$ From (5), we get $\kappa \left(M_{\mathrm{CFL}},d\right)\leq 12\left(2\right)=24$. Hence, by (2), we have $\alpha \left(M_{\mathrm{CFL}}\right)=\kappa \left(M_{\mathrm{CFL}}\right)=24.$*

*Finally, consider $N_{e}\left(d\right)=1$ and d=(1,1,1,1). The files $X_{2}$ and $X_{3}$ are not demanded by any user. In addition to the equations in E, here we consider a set of equations $X_{2,j}=0$ for j∈[12]. Thus there are 24 equations in total. Let S be the subspace of $F_{q}^{36}$ which satisfy these equations. By the rank-nullity theorem, the dimension of S is given by dim(S)≥36−24=12. The next step is to show that all the vectors in S lie in A(d). The all zero vector 0 lies in A(d) by definition. Any non-zero vector in S with $X_{1,j}\neq 0$ for j∈[12] lies in the corresponding $Z^{\left(i,j\right)}$. The 24 equations considered involve equations of the form $X_{2,j}=0$ for j∈[12]. Hence, by E, any $X_{3,j}\neq 0$ forces $X_{1,j}\neq 0$ for j∈[12] and hence such vectors also lie in A(d). Thus, all of the vectors in S lie in A(d). Therefore, $\alpha \left(M_{\mathrm{CFL}},d\right)\geq \dim\left(S\right)\geq 12.$ From (5), we get $\kappa \left(M_{\mathrm{CFL}},d\right)\leq 12\left(1\right)=12$. Hence, by (2), we have $\alpha \left(M_{\mathrm{CFL}}\right)=\kappa \left(M_{\mathrm{CFL}}\right)=12.$*

The theorem below gives the general expression for $\alpha (M_{\mathrm{CFL}},d)$, when N<K.

**Theorem** **2.** *For N<K and M=1/K,*

$$\begin{array}{cc} \alpha (M_{\mathrm{CFL}},d) & =\kappa (M_{\mathrm{CFL}},d)\\ & =\left\{\begin{array}{cc} NKN_{e}\left(d\right) & if\;N_{e}\left(d\right)\leq N-1\\ N^{2}\left(K-1\right) & if\;N_{e}\left(d\right)=N, \end{array}\right. \end{array}$$

*∀d∈D, where $N_{e}\left(d\right)$ is the number of distinct demands.*

**Proof.**  For N<K and M=1/K, the CFL prefetching scheme $M_{\mathrm{CFL}}$ is as follows. Each file is split into NK subfiles $X_{i}=\left(X_{i,1},X_{i,2},\ldots ,X_{i,NK}\right)$. User i,i∈[K] caches *N* coded packets given by $Y_{i}=X_{1,N(i-1)+j}⊕\ldots ⊕X_{N,N(i-1)+j},$ for j∈[N]. Let $X=(X_{1,1},\ldots ,X_{1,NK},\ldots ,$$X_{N,1},\ldots ,X_{N,NK})$ be the vector obtained by the concatenation of vectors $X_{i},i\in \left[N\right].$ For a given demand d, this problem becomes a generalized index coding problem $I(M_{\mathrm{CFL}},d)$ with $N^{2}K$ messages and $NK^{2}$ receivers.

First consider that all of the demands are distinct, i.e., $N_{e}\left(d\right)=N$. Without loss of generality we can assume that the first *N* users demand distinct files, such that the *i*th user demands $X_{d_{i}}$ for i=1,2,…,N. Thus, $d=(d_{1},d_{2},\ldots ,d_{K})$, such that $d_{i}\neq d_{j}$ for i,j∈[N]. Let $E=\{e_{i,j}:i\in \left[K\right],j\in \left[N\right]\}$ represent a set of NK equations, where $e_{i,j}:\left(X_{1,N(i-1)+j}⊕X_{2,N(i-1)+j}⊕\ldots ⊕X_{N,N(i-1)+j}\right)=0$. We consider a subset of the equations in *E* of the form $e_{i,j}$ for i,j∈[N]. There are $N^{2}$ such equations. Let S be the subspace of $F_{q}^{N^{2}K}$ consisting of vectors that satisfy these equations. From the rank-nullity theorem, we have dim$\left(S\right)\geq N^{2}K-N^{2}=N^{2}\left(K-1\right)$.

For $I(M_{\mathrm{CFL}},d)$, (1) can be rewritten as $Z^{\left(i,j\right)}≜\left\{z\in F_{q}^{N^{2}K}:e_{i,k}\;\mathrm{for}\;k\in \left[N\right],X_{d_{i},j}\neq 0\right\}$ for i∈[K] and j∈[NK]. Let $A\left(d\right)=\cup_{i\in [K],j\in [NK]}Z^{\left(i,j\right)}\cup \left\{0\right\}$. The generalized independence number is the maximum among the dimensions of all the subspaces of $F_{q}^{N^{2}K}$ in A(d). We show that S is such a subspace. For this, we need to show that all of the vectors of S lie in A(d). By the definition of A(d), the all zero vector 0 lies in A(d). The vectors that belong to S having $X_{d_{i},j}\neq 0$ belong to the set $Z^{\left(i,j\right)}$. Thus, all of the vectors in S lie in A(d) and $\alpha \left(M_{\mathrm{CFL}},d\right)\geq N^{2}\left(K-1\right).$ From (5), we get $\kappa \left(M_{\mathrm{CFL}}\right)\leq N^{2}\left(K-1\right)$. Hence, by (2), we have $\alpha \left(M_{\mathrm{CFL}}\right)=\kappa \left(M_{\mathrm{CFL}}\right)=N^{2}\left(K-1\right).$

Consider the case where $N_{e}\left(d\right)\leq N-1$. Let the first $N_{e}\left(d\right)$ demands be distinct and the *i*th user demands $X_{d_{i}}$ for $i\in [N_{e}\left(d\right)].$ Without loss of generality we can assume that the indices of the files that are not demanded are $N_{e}\left(d\right)+1,\ldots ,N$. There are $U=N-N_{e}\left(d\right)$ files that are not demanded. In addition to the NK equations that are presented in E, consider the following equations $X_{N_{e}\left(d\right)+i,j}=0$, for i∈[U−1] and j∈[NK]. The number of these equations is thus $NK+NK\left(U-1\right)=NKU=NK(N-N_{e}\left(d\right))$. Let S be the subspace of $F_{q}^{N^{2}K}$ which consists of the vectors satisfying these equations. By the rank-nullity theorem, dim$\left(S\right)\geq N^{2}K-NK\left(N-N_{e}\left(d\right)\right)=NK\left(N_{e}\left(d\right)\right).$ By definition, 0 lies in A(d). Any vector in S with the coordinate $X_{d_{i},j}\neq 0$ for $i\in [N_{e}\left(d\right)]$ lies in $Z^{\left(i,j\right)}$. The set of equations force all $X_{i,j}=0$ for $i\in \{N_{e}\left(d\right),\ldots ,N-1\}$ and j∈[NK]. Moreover, by the set of equations presented in E, $X_{N,j}\neq 0$ would mean some other $X_{d_{i},j}\neq 0$ for $i\in [N_{e}\left(d\right)]$. Hence, any vector with $X_{N,j}\neq 0$ lies in some $Z^{\left(i,j\right)}$ for $i\in [N_{e}\left(d\right)]$. Thus, all of the vectors in S lie in A(d). Therefore $\alpha \left(M_{\mathrm{CFL}},d\right)\geq \dim\left(S\right)\geq NKN_{e}\left(d\right).$ From (4), we have $\kappa \left(M_{\mathrm{CFL}},d\right)\leq NKN_{e}\left(d\right)$. Hence from (2), $\alpha \left(M_{\mathrm{CFL}},d\right)=\kappa \left(M_{\mathrm{CFL}},d\right)=NKN_{e}\left(d\right).$ □

## 4. Optimal Linear Error Correcting Delivery Scheme for the CFL Prefetching Scheme

In this section we give expressions for the average rate and the worst case rate for a δ-error correcting delivery scheme for the CFL prefetching scheme. Also we propose a δ-error correcting delivery scheme for this case. From Theorem 1 and Theorem 2, we can conclude that for all the generalized index coding problems $I(M_{\mathrm{CFL}},d)$ induced from the CFL prefetching scheme,

(6) $$\alpha \left(M_{\mathrm{CFL}},d\right)=\kappa \left(M_{\mathrm{CFL}},d\right).$$

Hence, the α and κ bounds in (3) meet. Using this, the optimal linear error correcting delivery scheme can be constructed for the CFL prefetching scheme and hence the average rate can be calculated, as given in the following theorem.

**Theorem** **3.** *For a coded caching problem with the CFL prefetching scheme for M=1/K,*

$$R^{*}\left(M_{\mathrm{CFL}},\delta \right)=E_{d}\left[\frac{N_{q}\left[\kappa \left(M_{\mathrm{CFL}},d\right),2\delta +1\right]}{n_{\mathrm{CFL}}}\right],$$

*where $n_{\mathrm{CFL}}$ is the number of subfiles into which each file is divided in the CFL scheme.*

**Proof.**  From (6) and (3), we can conclude that, for any generalized index coding problem induced from the coded caching problem with CFL prefetching, the α and κ bounds meet. Thus, the optimal linear error correcting delivery scheme would be the concatenation of the CFL delivery scheme with an optimal linear error correcting code. Thus, the optimal length for δ error corrections in those generalized index coding problems is $N_{q}\left[\kappa \left(M_{\mathrm{CFL}},d\right),2\delta +1\right]$ and, hence, the statement of the theorem follows. □

**Corollary** **1.** *For a coded caching problem with the CFL prefetching scheme for M=1/K,*

$$R_{\mathrm{worst}}^{*}\left(M_{\mathrm{CFL}},\delta \right)=\frac{N_{q}\left[\kappa_{\left\{\mathrm{worst}\right\}}\left(M_{\mathrm{CFL}},d\right),2\delta +1\right]}{n_{\mathrm{CFL}}},$$

*where the value of $\kappa_{\left\{\mathrm{worst}\right\}}\left(M_{\mathrm{CFL}},d\right)$ is obtained from (4) and (5) when $N_{e}\left(d\right)=N$.*

**Proof.**  Worst case rate is required when the number of distinct demands is maximum. This happens when $N_{e}\left(d\right)=N.$ □

Because the α and κ bounds become equal for $I(M_{\mathrm{CFL}},d)$, the optimal linear error correcting coded caching delivery scheme here would be the concatenation of the CFL delivery scheme with optimal classical error correcting scheme which corrects δ errors. Decoding can be done by syndrome decoding for error correcting generalized index codes proposed in [24,27].

In the remaining part of this section, few examples of the optimal linear error correcting delivery scheme for coded caching problems with the CFL prefetching are given.

**Example** **3.** *Consider the coded caching problem considered in Example 1 depicted in Figure 1. First consider that $N_{e}\left(d\right)=3$ and d=(1,2,3). We have shown that, for this case, $\kappa (M_{\mathrm{CFL}},d)=6$. The transmissions in the CFL scheme are $T_{1}:X_{2,1}$, $T_{2}:X_{3,1}$, $T_{3}:X_{1,2}$, $T_{4}:X_{3,2}$, $T_{5}:X_{1,3}$, and $T_{6}:X_{2,3}$. If δ=1 transmission error needs to be corrected, then, from [37], we have $N_{2}\left[6,3\right]=10$. A generator matrix that corresponds to ${\left[10,6,3\right]}_{2}$ code is*

$$G=\left[\begin{array}{cccccccccc} 1 & 0 & 0 & 0 & 0 & 0 & 1 & 1 & 0 & 0\\ 0 & 1 & 0 & 0 & 0 & 0 & 1 & 0 & 1 & 0\\ 0 & 0 & 1 & 0 & 0 & 0 & 1 & 0 & 0 & 1\\ 0 & 0 & 0 & 1 & 0 & 0 & 0 & 1 & 1 & 0\\ 0 & 0 & 0 & 0 & 1 & 0 & 0 & 1 & 0 & 1\\ 0 & 0 & 0 & 0 & 0 & 1 & 0 & 0 & 1 & 1 \end{array}\right].$$

*The optimal linear single error correcting delivery scheme is the concatenation of the CFL delivery scheme with the above code. Thus, single error correcting delivery scheme involves 10 transmissions. In addition to $T_{1},\ldots ,T_{6}$ the following transmissions are required.*

$$\begin{array}{cc} T_{7} & :X_{2,1}⊕X_{3,1}⊕X_{1,2},\\ T_{8} & :X_{2,1}⊕X_{3,2}⊕X_{1,3},\\ T_{9} & :X_{3,1}⊕X_{3,2}⊕X_{2,3}\;and\;\\ T_{10} & :X_{1,2}⊕X_{1,3}⊕X_{2,3}. \end{array}$$

*Now, consider $N_{e}\left(d\right)=2$ and d=(1,2,1). For this case also, $\kappa (M_{\mathrm{CFL}},d)=6$. The transmissions in the CFL scheme are $T_{1}:X_{1,1}$, $T_{2}:X_{1,2}$, $T_{3}:X_{1,3}$, $T_{4}:X_{2,1}$, $T_{5}:X_{2,2}$ and $T_{6}:X_{2,3}$. For single error correction, the concatenation is done with the same ${\left[10,6,3\right]}_{2}$ code. Considering the same generator matrix as before, the additional transmissions in the error correcting delivery scheme are*

$$\begin{array}{cc} T_{7} & :X_{1,1}⊕X_{1,2}⊕X_{1,3},\\ T_{8} & :X_{1,1}⊕X_{2,1}⊕X_{2,2},\\ T_{9} & :X_{1,2}⊕X_{2,1}⊕X_{2,3}\;and\;\\ T_{10} & :X_{1,3}⊕X_{2,2}⊕X_{2,3}. \end{array}$$

*Finally, consider $N_{e}\left(d\right)=1$ and d=(1,1,1). For this case, $\kappa (M_{\mathrm{CFL}},d)=3$. The CFL transmission scheme involves the following three transmissions $T_{1}:X_{1,1}$, $T_{2}:X_{1,2}$, and $T_{3}:X_{1,3}$. For single error correction, we have from [37] that $N_{2}\left[3,3\right]=6$. A generator matrix for the ${\left[6,3,3\right]}_{2}$ code is*

$$G=\left[\begin{array}{cccccc} 1 & 0 & 0 & 1 & 1 & 0\\ 0 & 1 & 0 & 1 & 0 & 1\\ 0 & 0 & 1 & 0 & 1 & 1 \end{array}\right].$$

*Thus, the optimal linear single error correcting delivery scheme is the concatenation of the CFL delivery scheme with the above code. The additional transmissions required apart from $T_{1},T_{2}$ and $T_{3}$ are*

$$\begin{array}{cc} T_{4} & :X_{1,1}⊕X_{1,2},\\ T_{5} & :X_{1,1}⊕X_{1,3}\;and\;\\ T_{6} & :X_{1,2}⊕X_{1,3}. \end{array}$$

*Decoding is done by syndrome decoding for generalized index codes [24,27].*

**Example** **4.** *Consider the coded caching problem considered in Example 2 depicted in Figure 2. Consider that $N_{e}\left(d\right)=3$ and d=(1,2,3,1). We have shown that for this case $\kappa (M_{\mathrm{CFL}},d)=27$. The transmissions in the CFL scheme are $T_{1}:X_{2,1}$, $T_{2}:X_{3,1}$, $T_{3}:X_{2,2}$, $T_{4}:X_{3,2}$, $T_{5}:X_{2,3}$, $T_{6}:X_{3,3}$, $T_{7}:X_{1,4}$, $T_{8}:X_{3,4}$, $T_{9}:X_{1,5}$, $T_{10}:X_{3,5}$, $T_{11}:X_{1,6}$, $T_{12}:X_{3,6}$, $T_{13}:X_{1,7}$, $T_{14}:X_{2,7}$, $T_{15}:X_{1,8}$, $T_{16}:X_{2,8}$, $T_{17}:X_{1,9}$, $T_{18}:X_{2,9}$, $T_{19}:X_{2,10}$, $T_{20}:X_{3,10}$, $T_{21}:X_{2,11}$, $T_{22}:X_{3,11}$, $T_{23}:X_{2,12}$, $T_{24}:X_{3,12}$, $T_{25}:X_{1,1}⊕X_{1,10}$, $T_{26}:X_{1,2}⊕X_{1,11}$, and $T_{27}:X_{1,3}⊕X_{1,12}$. If δ=1 transmission error needs to be corrected, then from [37], we have $N_{2}\left[27,3\right]=42$. The optimal linear single error correcting delivery scheme involves concatenation of CFL delivery scheme with a generator matrix that corresponds to the ${\left[42,27,3\right]}_{2}$ code.*

## 5. Optimal Linear Error Correcting Scheme for M=1/K with Reduced Sub-Packetization

In this section, we consider the scheme presented in [4], which we call the JV scheme for M=1/K and discuss the error correction. For the case N=K, the JV scheme is exactly same as the CFL scheme. For K>N, the JV scheme has an advantage in terms of sub-packetization. The JV scheme splits each file into *K* subfiles where as CFL scheme required NK subfiles. Here, we find a closed form expression for the generalized independence number $\alpha (M_{\mathrm{JV}},d)$ of the index coding problem $I(M_{\mathrm{JV}},d)$. For all of the index coding problems that correspond to all possible demands, we show that the generalized independence number is equal to the min-rank. Hence in this case also, α and κ bounds meet and the optimal error correcting delivery scheme is obtained by concatenation scheme.

The expression for min-rank for this scheme can be obtained from the achievable rate expressions in [3,4], as

(7) $$\kappa \left(M_{\mathrm{JV}},d\right)\leq \left\{\begin{array}{cc} KN_{e}\left(d\right) & \mathrm{if}\;N_{e}\left(d\right)\leq N-1\\ N(K-1) & \mathrm{if}\;N_{e}\left(d\right)=N \end{array}\right..$$

The calculation of generalized independence number and how it becomes equal to the min-rank are illustrated in the following example.

**Example** **5.** *Consider a coded caching problem with N=3, K=6 and M=1/6. According to the JV scheme, each file is split into K=6 subfiles as: $X_{i}=\left(X_{i,1},X_{i,2},X_{i,3},X_{i,4},X_{i,5},X_{i,6}\right)$ for i∈{1,2,3}. Let X be the vector obtained by concatenation of $X_{1}$, $X_{2}$ and $X_{3}$, given by $X=(X_{1,1},\ldots ,X_{1,6},X_{2,1},\ldots ,X_{2,6},X_{3,1},\ldots ,X_{3,6})$. The cache contents of user i is $Y_{i}=X_{1,i}⊕X_{2,i}⊕X_{3,i}$ for i∈{1,2,…,6}. For a given demand d, this problem becomes a generalized index coding problem $I(M_{\mathrm{JV}},d)$, having 18 messages and 36 receivers.*

*First consider that $N_{e}\left(d\right)=N=3$ and let d=(1,2,3,1,2,3). Consider the equations $e_{i}:X_{1,i}⊕X_{2,i}⊕X_{3,i}=0$ for i∈{1,2,…,6}. Out of these, consider the first three equations $e_{1},e_{2}$ and $e_{3}$. Let S be the subspace of $F_{q}^{18}$ consisting of vectors which satisfy these three equations. Subsequently, from the rank-nullity theorem, we have dim(S)≥18−3=15. For this case, (1) can be rewritten as $Z^{\left(i,j\right)}≜\left\{z\in F_{q}^{18}:e_{i},X_{d_{i},j}\neq 0\right\}$ for i,j∈[6]. Let $A\left(d\right)=\cup_{i\in [6],j\in [6]}Z^{\left(i,j\right)}\cup \left\{0\right\}$. The generalized independence number is the maximum among the dimensions of all the subspaces of $F_{q}^{18}$ in A(d). We claim that S is such a subspace. This would mean that $\alpha \left(M_{\mathrm{JV}},d\right)\geq \dim\left(S\right)\geq 15$. For this, we need to show that all of the vectors in S lie in A(d). By the definition of A(d), the all zero vector 0 lies in A(d). Any other vector in S will have at least one non-zero coordinate. All of the vectors in S, having $X_{d_{i},j}\neq 0$ belongs to $Z^{\left(i,j\right)}$. Because all files are demanded, all of the vectors in S lie in A(d) and $\alpha (M_{\mathrm{JV}},d)\geq 15.$ From (7), we get $\kappa \left(M_{\mathrm{JV}},d\right)\leq 3\left(6-1\right)=15$. Hence, by (2), we have $\alpha \left(M_{\mathrm{JV}},d\right)=\kappa \left(M_{\mathrm{JV}},d\right)=15.$*

*Consider now that $N_{e}\left(d\right)=2$ and d=(1,2,1,2,1,2). In this case, consider the six equations $e_{1},e_{2},\ldots ,e_{6}$. Let S be the subspace of $F_{q}^{18}$ consisting of vectors which satisfy these equations. Hence from the rank-nullity theorem, we have dim(S)≥18−6=12. By definition, 0 lies in A(d). Any non-zero vector in S with $X_{d_{i},j}\neq 0$ for i=1,2 and j=1,2,…,6 lies in the corresponding $Z^{\left(i,j\right)}$. Now, we have to consider the vectors that satisfy these equations and $X_{3,j}\neq 0$ for j=1,2,…,6. From the equations $e_{i}$, any $X_{3,j}\neq 0$ forces some $X_{i,j}\neq 0$ for i=1,2 and, hence, such vectors also lie in A(d). Thus, all of the vectors in S lie in A(d). Therefore, $\alpha \left(M_{\mathrm{JV}},d\right)\geq \dim\left(S\right)\geq 12.$ From (7), we get $\kappa \left(M_{\mathrm{JV}},d\right)\leq 6\left(2\right)=12$. Hence, by (2), we have $\alpha \left(M_{\mathrm{JV}},d\right)=\kappa \left(M_{\mathrm{JV}},d\right)=12.$*

*Finally, consider $N_{e}\left(d\right)=1$ and d=(1,1,1,1,1,1). The files $X_{2}$ and $X_{3}$ are not demanded by any user. Here, we consider the equation $e_{1},e_{2},\ldots ,e_{6}$. In addition to this, consider the equations $X_{2,1}=0$, $X_{2,2}=0$, $X_{2,3}=0$, $X_{2,4}=0$, $X_{2,5}=0$ and $X_{2,6}=0$. Thus, there are twelve equations in total. Let S be the subspace of $F_{q}^{18}$ which satisfy these equations. By the rank-nullity theorem, the dimension of S is given by dim(S)≥18−12=6. The next step is to show that all of the vectors in S lie in A(d). The all zero vector 0 lies in A(d) by definition. Any non-zero vector in S with $X_{1,j}\neq 0$ for j∈[6] lies in the corresponding $Z^{\left(1,j\right)}$. From the set of twelve equations, $X_{3,j}\neq 0$ force some $X_{1,j}\neq 0$ for j∈[6] and, hence, such vectors also lie in A(d). Thus, all of the vectors in S lie in A(d). Therefore, $\alpha \left(M_{\mathrm{JV}},d\right)\geq \dim\left(S\right)\geq 6.$ From (7), we get $\kappa \left(M_{\mathrm{JV}},d\right)\leq 6\left(1\right)=6$. Hence, by (2), we have $\alpha \left(M_{\mathrm{JV}},d\right)=\kappa \left(M_{\mathrm{JV}},d\right)=6.$*

This can be generalized as in the theorem below.

**Theorem** **4.** *For N<K and M=1/K,*

$$\alpha \left(M_{\mathrm{JV}},d\right)=\kappa \left(M_{\mathrm{JV}},d\right)=\left\{\begin{array}{cc} KN_{e}\left(d\right) & if\;N_{e}\left(d\right)\leq N-1\\ N(K-1) & if\;N_{e}\left(d\right)=N, \end{array}\right.$$

*∀d∈D, where $N_{e}\left(d\right)$ is the number of distinct demands.*

**Proof.**  For N<K and M=1/K, the JV prefetching scheme $M_{\mathrm{JV}}$ is as follows. Each file is split into *K* subfiles $X_{i}=\left(X_{i,1},X_{i,2},\ldots ,X_{i,K}\right)$. The cache content of user *i* is given as $Y_{i}=X_{1,i}⊕X_{2,i}⊕\ldots X_{N,i}$. Let $X=(X_{1,1},\ldots ,X_{1,K},X_{2,1},\ldots ,X_{2,K},X_{3,1}\ldots ,$$X_{N,1},\ldots ,X_{N,K})$ be the vector obtained by the concatenation of vectors $X_{i},i\in \left[N\right].$ For a given demand d, this problem becomes a generalized index coding problem $I(M_{\mathrm{JV}},d)$ with NK messages and $K^{2}$ receivers.

First, consider that all of the demands are distinct, i.e., $N_{e}\left(d\right)=N$. Without a loss of generality, we can assume that the first *N* users demand distinct files, such that $X_{d_{i}}=X_{i}$ for i=1,2,…,N. Thus $d=(d_{1},d_{2},\ldots ,d_{K})$, such that $d_{i}=i$ for i,j∈[N]. Consider the set of equations $e_{i}:X_{1,i}⊕X_{2,i}⊕\ldots X_{N,i}=0$ for i∈[K]. Out of these equations, consider a subset of *N* equations $e_{i}$ for i∈[N]. Let S be the subspace of $F_{q}^{NK}$ consisting of vectors satisfying these equations. From the rank-nullity theorem, we have dim(S)≥NK−N=N(K−1).

For $I(M_{\mathrm{JV}},d)$, (1) can be rewritten as $Z^{\left(i,j\right)}≜\left\{z\in F_{q}^{NK}:e_{i}\;\mathrm{for}\;i\in \left[N\right],X_{d_{i},j}\neq 0\right\}$ for i,j∈[K]. Let $A\left(d\right)=\cup_{i\in [K],j\in [K]}Z^{\left(i,j\right)}\cup \left\{0\right\}$. The generalized independence number is the maximum among the dimensions of all the subspaces of $F_{q}^{NK}$ in A(d). We show that S is such a subspace. For this, we need to show that all the vectors of S lie in A(d). By the definition of A(d), the all zero vector 0 lies in A(d). The vectors that belong to S having $X_{d_{i},j}\neq 0$ belong to the set $Z^{\left(i,j\right)}$. Thus, all of the vectors in S lie in A(d) and $\alpha \left(M_{\mathrm{JV}},d\right)\geq N\left(K-1\right).$ From (7), we get $\kappa \left(M_{\mathrm{JV}}\right)\leq N\left(K-1\right)$. Hence, by (2), we have $\alpha \left(M_{\mathrm{JV}},d\right)=\kappa \left(M_{\mathrm{JV}},d\right)=N\left(K-1\right).$

Consider the case where $N_{e}\left(d\right)\leq N-1$. Let the first $N_{e}\left(d\right)$ demands be distinct and without loss of generality assume that the *i*th user demands $X_{i}$ for $i\in [N_{e}\left(d\right)].$ Thus, the indices of the files that are not demanded are $N_{e}\left(d\right)+1,\ldots ,N$. There are $U=N-N_{e}\left(d\right)$ files that are not demanded. In addition to the *K* equations $e_{i}$, consider the following equations $X_{i,j}=0$, for $i\in \{N_{e}\left(d\right)+1,\ldots ,N-1\}$ and j∈[K]. Thus, the number of these equations is $K+K\left(U-1\right)=KU=K(N-N_{e}\left(d\right))$. Let S be the subspace of $F_{q}^{NK}$ which consists of the vectors satisfying these equations. By the rank-nullity theorem, dim$\left(S\right)\geq NK-K\left(N-N_{e}\left(d\right)\right)=KN_{e}\left(d\right).$ By definition, 0 lies in A(d). Any vector in S with the coordinate $X_{i,j}\neq 0$ for $i\in [N_{e}\left(d\right)]$ lies in $Z^{\left(i,j\right)}$. The set of equations force all $X_{i,j}=0$ for $i\in \{N_{e}\left(d\right),\ldots ,N-1\}$ and j∈[K]. Moreover by the same set of equations, $X_{N,j}\neq 0$ would mean some other $X_{i,j}\neq 0$ for $i\in [N_{e}\left(d\right)]$. Hence any vector with $X_{N,j}\neq 0$ lies in some $Z^{\left(i,j\right)}$ for $i\in [N_{e}\left(d\right)]$. Thus all the vectors in S lie in A(d). Therefore, $\alpha \left(M_{\mathrm{JV}},d\right)\geq \dim\left(S\right)\geq KN_{e}\left(d\right).$ From (7), we have $\kappa \left(M_{\mathrm{JV}},d\right)\leq KN_{e}\left(d\right)$. Hence, from (2), $\alpha \left(M_{\mathrm{JV}},d\right)=\kappa \left(M_{\mathrm{JV}},d\right)=NKN_{e}\left(d\right).$ □

Because, for this scheme, also, α and κ bounds meet, the optimal coded caching delivery scheme here would be the concatenation of the JV delivery scheme with optimal classical error correcting scheme that corrects δ errors. Decoding can be done by syndrome decoding for error correcting generalized index codes proposed in [24,27]. This is illustrated while using an example below.

**Example** **6.** *Consider the coded caching problem considered in Example 5. Consider that $N_{e}\left(d\right)=3$ and d=(1,2,3,1,2,3). We have shown that for this case $\kappa (M_{\mathrm{JV}},d)=15$. The transmissions in the JV scheme are $T_{1}:X_{1,2}$, $T_{2}:X_{1,3}$, $T_{3}:X_{1,5}$, $T_{4}:X_{1,6}$, $T_{5}:X_{1,4}⊕X_{1,1}$, $T_{6}:X_{2,1}$, $T_{7}:X_{2,3}$, $T_{8}:X_{2,4}$, $T_{9}:X_{2,6}$, $T_{10}:X_{2,5}⊕X_{2,2}$, $T_{11}:X_{3,1}$, $T_{12}:X_{3,2}$, $T_{13}:X_{3,4}$, $T_{14}:X_{3,5}$, $T_{15}:X_{3,6}⊕X_{3,3}$. If δ=1 transmission error needs to be corrected, then from [37], we have $N_{2}\left[15,3\right]=20$. The optimal linear single error correcting delivery scheme involves a concatenation of JV delivery scheme with a generator matrix that corresponds to the ${\left[20,15,3\right]}_{2}$ code.*

## 6. Conclusions

In this work, we obtained the minimum number of transmissions required for a linear δ-error correcting delivery scheme for coded caching problems with the CFL prefetching scheme. We proposed an optimal linear error correcting delivery scheme for the above case. We also found closed form expressions for the average rate and the peak rate for these problems. We considered the JV scheme, which uses low sub-packetization as compared to CFL scheme and proved that for this scheme also concatenation of JV delivery scheme with optimal error correcting code is optimal.

## Figures and Tables

**Table 1:** Generalized independence number and min-rank of $I(M_{\mathrm{CFL}},d)$ for different d in Example 1.

**d**	$\alpha (M_{\mathrm{CFL}},d)$	$\kappa (M_{\mathrm{CFL}},d)$	**d**	$\alpha (M_{\mathrm{CFL}},d)$	$\kappa (M_{\mathrm{CFL}},d)$	**d**	$\alpha (M_{\mathrm{CFL}},d)$	$\kappa (M_{\mathrm{CFL}},d)$
(1,2,3)	6	6	(1,3,3)	6	6	(2,2,3)	6	6
(1,3,2)	6	6	(3,1,3)	6	6	(2,3,2)	6	6
(2,1,3)	6	6	(3,3,1)	6	6	(3,2,2)	6	6
(2,3,1)	6	6	(1,1,2)	6	6	(2,3,3)	6	6
(3,1,2)	6	6	(1,2,1)	6	6	(3,2,3)	6	6
(3,2,1)	6	6	(2,1,1)	6	6	(3,3,2)	6	6
(1,2,2)	6	6	(1,1,3)	6	6	(1,1,1)	3	3
(2,1,2)	6	6	(1,3,1)	6	6	(2,2,2)	3	3
(2,2,1)	6	6	(3,1,1)	6	6	(3,3,3)	3	3
